# Health‐related quality of life in cutaneous T‐cell lymphoma: A cross‐sectional survey study

**DOI:** 10.1002/ski2.45

**Published:** 2021-05-15

**Authors:** M.M. Shinohara, H.M. Mahurin, E. Tarabadkar, D.S. Hippe, K. Lachance, E.J. Kim, E.T. Loggers

**Affiliations:** ^1^ Division of Dermatology University of Washington Seattle Washington USA; ^2^ School of Medicine University of Washington Seattle Washington USA; ^3^ Department of Dermatology Emory University Atlanta Georgia USA; ^4^ Department of Radiology University of Washington Seattle Washington USA; ^5^ Department of Dermatology University of Pennsylvania Philadelphia Pennsylvania USA; ^6^ Clinical Research Division Fred Hutchinson Cancer Research Center Seattle Washington USA; ^7^ Division of Hematology/Oncology Department of Medicine University of Washington Seattle Washington USA

## Abstract

**Background:**

Patients with cutaneous T‐cell lymphoma (CTCL) often have indolent but symptomatic disease.

**Objective:**

Assessment of the health‐related quality of life (HRQoL) of patients with CTCL.

**Methods:**

Cross‐sectional survey study. HRQoL was measured by Skindex‐16 and FACT‐G.

**Results:**

A total of 372 responses were received; 80 incomplete/ineligible responses were excluded. A majority of respondents identified as white (87%; 250/288) and female (67%; 193/286) with a mean age of 57 ± 14 years. Most patients had early‐stage (IA–IIA) (74%; 162/203) mycosis fungoides (87%; 241/279). There were 33 (12%; 33/279) patients with Sézary syndrome. Mean itch score (visual analogue scale; VAS) was 3.2 ± 2.8, overall; 2.7 ± 2.6 for early, and 4.2 ± 2.9 for advanced disease (*p* = 0.008). Thirty‐eight percent (108/284) and 24% (69/284) reported head/neck and groin/genital involvement, respectively.

Overall HRQoL was 46 ± 27 (Skindex‐16) and 71 ± 19 (FACT‐G), with worse HRQoL for patients with advanced versus early disease (Skindex‐16: 67 vs. 40; *p*=<0.001, FACT‐G: 62 vs. 76; *p* = 0.001). Predictors of worse HRQoL included head/neck, hand/foot or groin/genital involvement, younger age and spending >15 min daily treating CTCL.

**Limitations:**

Include anonymous survey methodology, underrepresentation of certain CTCL subtypes and non‐white respondents.

**Conclusions:**

Patients with CTCL, particularly those with advanced disease or involvement of the head/neck, acral or groin/genital sites, experience significant impact on HRQoL.

## Graphical abstract

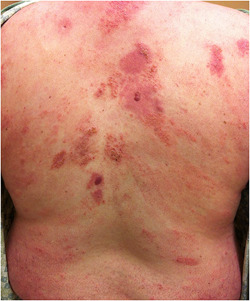

1

**What is already known about this topic?**

- Cutaneous T‐cell lymphoma (CTCL) is a cancer and chronic skin disease. Patients often have indolent but symptomatic disease.

**What does this study add?**

- Patients with CTCL experience suffering across multiple domains, including their sexual lives. Evaluating for and treating CTCL in the head/neck, acral and groin/genital areas could significantly impact patient’s overall health‐related quality of life.

## 2 INTRODUCTION

Most patients with cutaneous T‐cell lymphoma (CTCL) live with their disease for years or even decades. Symptom burden in CTCL can be significant, with as many as 88% reporting pruritus in the previous month.^1^ A growing body of literature supports the impact of skin disease on health‐related quality of life (HRQoL). Chronic skin diseases can cause significant psychological and social distress such as depression and fear of stigma,^2^ while the disability experienced by patients with psoriasis is comparable to that of chronic illnesses such as heart disease and diabetes.^3^ Cancer also negatively impacts HRQoL in patients, even for survivors.^4^ For example, survivors of non‐Hodgkin lymphoma have worse HRQoL compared to age and sex‐matched normative controls.^5^, ^6^

The Cutaneous Lymphoma Foundation (CLF) is an independent, non‐profit patient advocacy organization dedicated to supporting people affected by cutaneous lymphoma (https://www.clfoundation.org/about‐us). A large‐scale study done in 2005 by Demierre et al. in partnership with the CLF found that CTCL has a profound impact on HRQoL, with worse HRQoL reported for more advanced disease.^7^ We partnered with the CLF to examine QoL in patients with CTCL, with a focus on physical, social and emotional well‐being. We hypothesized that despite advances in CTCL therapy since the work of Demierre et al. more than 15 years ago, patients with CTCL still experience lower HRQoL. We also examined the impact of disease stage, location of skin involvement, time since diagnosis and itch severity on HRQoL for those with CTCL.

## 3 MATERIALS AND METHODS

A cross‐sectional, anonymous electronic survey was administered between February and April 2019. The survey was distributed via a link posted on the CLF Facebook page and email lists. At the time of distribution, the CLF Facebook group had approximately 1900 members, while the email listerv had approximately 1200 members. The study team did not directly access membership lists. Survey responses were collected and managed using REDCap electronic data capture tools hosted at the University of Washington.^8^, ^9^ This study was determined to be exempt from institutional review board review by the University of Washington Human Subjects Division (STUDY00005784).

Independent measures included demographics, CTCL type and stage, time since diagnosis, and comorbid conditions (adapted from the self‐reported Charlson Comorbidity Index^10^). Four independent measures, three of which were similar to those in Demierre's original study and one novel question, assessed the emotional and financial burden of CTCL (collectively termed the ‘Burden Score’). Itch was measured using VAS. Two validated HRQoL instruments were included: Skindex‐16^11^ and Functional Assessment of Cancer Therapy: General (FACT‐G).^12^ Skindex‐16 scores were calculated per Chren et al.,^11^ transforming responses to a linear scale from 0 to 100. Total score and subscale scores were considered valid if at least 70% of the items had responses. Missing items in each subscale were imputed with the mean of the non‐missing items in that subscale. FACT‐G scores were calculated according to the FACT‐G Scoring Guidelines Version 4. Scores were calculated for each subscale and summed together to derive the total score, with a range of 0–108 (with lower scores indicating worse HRQoL). Subscale scores were considered valid if >50% of the items had responses (i.e., ≥4 of 6 or 7 items per subscale). The total score was considered valid if >80% (≥22 of 27 items) had responses and all subscales were valid. Missing items in each subscale were imputed using the mean of non‐missing items in that subscale.

HRQoL scores and demographic variables were compared between these groups using the Wilcoxon rank‐sum test or Fisher’s exact test. Linear regression models were used to examine mean differences in Skindex‐16 or FACT‐G. *p*‐Values less than 0.05 were considered statistically significant. *Β*‐values are mean differences in Skindex‐16 or FACT‐G per change in the corresponding variable; estimates were derived using linear regression models. All statistical analyses were performed using STATA (version 14.0; StataCorp) and R software (version 4.0.0; R Foundation for Statistical Computing).

## 4 RESULTS

A total of 372 responses were received. Response rate could not be calculated due to the survey distribution methods, but is estimated between 12% and 30% based on the maximum (3100) and minimum (1240) number of patients who could have viewed the survey link. Seventy‐three respondents who stopped prior to the end of the survey (19.6%) and seven (1.7%) additional responses from patients with cutaneous B‐cell lymphoma (CBCL) were excluded from the final analysis, leaving 292 participants. Demographics of this cohort are summarized in Table 1. A majority identified as white (87%; 250/288) and female (66%; 190/286). Respondent age ranged from 24 to 92 years, with a mean of 57 ± 14 years. Among all respondents, 76% (209/276) had completed at least an undergraduate degree, and most had commercial insurance (59%; 174/290) followed by Medicare (34%; 99/290). The most common reported medical comorbidity of the cohort was bone or joint problems (16%; 48/292). Diabetes mellitus was the second most common (10%; 30/292), while chronic obstructive pulmonary disease and heart disease each affected approximately 5% of the cohort.

**TABLE 1:** Demographics of study cohort (*N* = 292 unless otherwise indicated)

Variable	Number (%^a^) (or Mean ± SD)
Gender	
Female	190 (66.2)
Male	95 (33.1)
Other	2 (0.7)
Age, mean (range 24–92 years)	57 ± 14
Race/ethnicity	
Asian	2 (0.7)
American Indian or Alaska Native	2 (0.7)
Black or African American	12 (4.2)
Hispanic or Latino	12 (4.2)
Native Hawaiian or other Pacific Islander	0 (0.0)
White	250 (86.8)
Other	7 (2.4)
Multiple races	3 (1.0)
Married or in a domestic partnership	208 (72.0)
Location	
Inside United States	213 (75.3)
Outside United States	70 (24.7)
Education	
High school or less	67 (24.3)
Associate's or Bachelor's degree	130 (47.1)
Graduate or professional degree	79 (28.6)
Employment	
Disabled	23 (7.7)
Employed	130 (45.5)
Student	6 (2.1)
Retired	118 (41.3)
Unemployed	11 (3.8)
Work from home/homemaker	22 (7.7)
Health insurance/coverage status	
Commercial insurance	174 (59.2)
Medicare	99 (33.7)
Other	43 (14.6)
Medicaid	17 (5.8)
No insurance or coverage	17 (5.8)
Veterans Affairs or Tricare	9 (3.1)
Charity care	2 (0.7)
Self‐reported comorbid medical condition^b^	
None	189 (64.7)
1 or more	103 (35.2)
Time since diagnosis	
<1 year	63 (21.6)
1–5 years	126 (43.2)
>5 years	103 (35.3)
Type of CTCL (multiple options)	
Mycosis fungoides (MF)	241 (86.7)
Sézary syndrome (SS)	33 (11.9)
pcALCL or LyP	26 (9.4)
Other^c^	2 (0.7)
Stage, MF/SS	
Early (IA–IIA)	162 (79.8)
Advanced (IIB–IVB)	41 (20.2)
Itch score (VAS)	3.2 ± 2.8
Early (IA–IIA)	2.7 ± 2.6
Advanced (IIB–IVB)	4.2 ± 2.9
Areas of body involved (multiple options)	
Legs	186 (65.5)
Arms	168 (59.2)
Chest or sides	163 (57.4)
Back	150 (52.8)
Buttocks	157 (55.3)
Head/neck	108 (38.0)
Feet	98 (34.5)
Hands	78 (27.5)
Groin/genitals	69 (24.3)

*Notes:* Total number may not equal 292 due to incomplete responses. *p* = 0.008.

Abbreviations: CTCL, cutaneous T‐cell lymphoma; LyP, lymphomatoid papulosis; pcALCL, primary cutaneous anaplastic large cell lymphoma; SD, standard deviation.

^a^ Respondents who did not provide a value were excluded from the corresponding summary: gender (*n* = 5), age (*n* = 9), race/ethnicity (*n* = 4), marital status (*n* = 3), location (*n* = 9), education (*n* = 16), employment (*n* = 6), insurance status (*n* = 1), type of CTCL (*n* = 14), stage (*n* = 73), itch score (*n* = 8) and area of body involved (*n* = 8).

^b^ Includes items from the Charleston Comorbidity Index: myocardial infarction; heart failure; peripheral vascular disease; chronic obstructive lung disease; emphysema; stomach ulcers; liver disease; hepatitis; stroke/mini‐stroke; hemiplegia; dementia; rheumatoid arthritis; lupus, scleroderma, Sjögren’s, or connective tissue disease; other joint/bone problems; series kidney problems; and diabetes.

^c^ Other types of CTCL = subcutaneous panniculitis‐like T‐cell lymphoma, primary cutaneous peripheral T‐cell lymphoma.

The majority (65%; 189/292) of patients had been diagnosed with their CTCL within the previous 5 years. Mycosis fungoides (MF) was the most common type of CTCL (87%; 241/278), followed by Sézary syndrome (SS) (12%; 33/278) and CD30 lymphoproliferative disorders (LPDs) (9%; 26/278). Among patients with MF/SS who reported their stage (*n* = 203, 70%), the majority (80%; 162/203) had early‐stage disease (IA–IIA). The extremities (legs/arms), trunk and buttocks were the most commonly reported sites of involvement (Table 1); 38% (108/284) reported involvement of the head/neck, and 24% (69/284) reported involvement of the groin/genitals. The mean itch score (VAS) of the entire cohort was 3.2 ± 2.8, 2.7 ± 2.6 for those with early‐stage MF/SS, and 4.2 ± 2.9 for those with late‐stage MF/SS (*p* = 0.008). Results from the Burden Score items are reported in Table 2.

**TABLE 2:** Percent of participants responding positively (sometimes, often or very often) to Burden Score items (*N* = 292)

	Number (%^a^)
I have been treated unfairly by service establishments (such as hair salons/barbers, public pools, health clubs, etc.) because of my cutaneous lymphoma.	32/287 (11.1)
My cutaneous lymphoma has been mistaken for a contagious condition.	84/286 (29.4)
I feel financially burdened by the cost of managing my cutaneous lymphoma.	165/291 (57.1)
I feel I have to keep my cutaneous lymphoma private from others (including work or friends).	162/289 (55.7)
One or more of the above	245/286 (85.7)

^a^ Multiple selections possible.

The majority (74%; 215/289) of patients reported spending <60 min daily treating their CTCL (including application of topical treatments, appointments, infusions), with most (38%; 109/289) spending <15 min daily and 26% (74/289) spending >60 min daily. 15% (42/289) spend over 2 h daily. Over half (52%; 152/292) of the respondents indicated that they received help from a caregiver or family member for their CTCL.

Mean overall Skindex‐16 and FACT‐G scores and scores for MF/SS by stage are shown in Table 3. Association of HRQoL as measured by Skindex‐16 and FACT‐G is shown in Tables 4 and 5, respectively. Age, advanced stage, higher itch scores by VAS, head/neck, groin/genital or acral involvement were all significantly associated with worse HRQoL in both instruments, as were requiring help from a caregiver, spending >15 min daily treating CTCL (vs < 15 min daily), each of the independent measures of the Burden Score and the overall Burden Score. Time since diagnosis of CTCL (within 1 year vs. longer) was not significantly associated with HRQoL by either instrument. Patients who reported groin/genital involvement had lower satisfaction with their sex life as assessed by FACT‐G, with 46% (25/54) of patients with groin/genital involvement reporting they are ‘not at all’ satisfied with their sex life compared to 25% (41/161) of patients with other body areas involved (*p* = 0.006) (data not included in tables).

**TABLE 3:** Health‐related quality of life by Skindex‐16 and FACT‐G by stage (MF/SS)

	All	MF/SS by stage		*p*‐Value
		Early (IA–IIA)	Advanced (IIB–IVB)	
Skindex‐16				
Total score	46 ± 27	42 ± 26	59 ± 23	<0.001
Symptoms subscale	39 ± 30	33 ± 27	49 ± 32	0.004
Emotions subscale	58 ± 29	55 ± 30	71 ± 23	0.004
Function subscale	35 ± 32	30 ± 30	51 ± 29	<0.001
FACT‐G				
Total score	71 ± 19	74 ± 19	63 ± 19	0.001
PWB subscale	20 ± 6	22 ± 6	18 ± 7	<0.001
SWB subscale	19 ± 7	18 ± 7	18 ± 6	0.69
EWB subscale	15 ± 6	15 ± 6	13 ± 5	0.088
FWB subscale	18 ± 6	19 ± 6	14 ± 6	<0.001

*Note:* Values are mean ± SD unless otherwise specified.

Abbreviations: EWB, emotional well‐being; FWB, functional well‐being; MF, mycosis fungoides; PWB, physical well‐being; SS, Sézary syndrome; SWB, social/family well‐being.

**TABLE 4:** Associations with Health‐Related Quality of Life as measured by the Skindex‐16

	Total score		Symptoms subscale		Emotions subscale		Function subscale	
Variable	β^a^ (95% CI)	*p*‐Value	β^a^ (95% CI)	*p*‐Value	β^a^ (95% CI)	*p*‐Value	β^a^ (95% CI)	*p*‐Value
Age, per 10‐year increase	−2.6 (−4.8, −0.3)	0.028	−0.0 (−2.5, 2.5)	0.98	−3.5 (−5.9, −1.0)	0.006	−3.6 (−6.3, −0.9)	0.009
Advanced stage (vs. early stage)	17.5 (8.8, 26.3)	<0.001	16.0 (6.4, 25.5)	0.001	15.5 (5.5, 25.4)	0.002	21.3 (11.0, 31.6)	<0.001
Body area involved (vs. other area)								
Head and neck	19.6 (13.6, 25.7)	<0.001	20.6 (14.0, 27.3)	<0.001	15.9 (9.1, 22.7)	<0.001	23.8 (16.6, 31.0)	<0.001
Hands or feet	14.9 (8.7, 21.0)	<0.001	17.9 (11.2, 24.7)	<0.001	11.6 (4.8, 18.5)	0.001	16.3 (8.9, 23.7)	<0.001
Groin	16.7 (9.7, 23.8)	<0.001	21.7 (14.0, 29.3)	<0.001	12.4 (4.5, 20.2)	0.002	17.7 (9.3, 26.2)	<0.001
Itching severity, per 1‐point increase	5.5 (4.6, 6.4)	<0.001	7.9 (7.1, 8.7)	<0.001	4.4 (3.3, 5.5)	<0.001	5.3 (4.1, 6.4)	<0.001
Time since diagnosis		0.21		0.19		0.31		0.22
<1 year	(ref)		(ref)		(ref)		(ref)	
1–5 years	−2.2 (−10.5, 6.1)		0.9 (−8.1, 9.9)		−4.8 (−13.8, 4.3)		−2.1 (−11.9, 7.6)	
>5 years	−4.4 (−12.9, 4.2)		−2.8 (−12.2, 6.5)		−5.8 (−15.2, 3.5)		−4.5 (−14.7, 5.6)	
Help from caregiver (vs. no help)	7.6 (1.4, 13.8)	0.016	4.9 (−1.9, 11.7)	0.16	6.5 (−0.3, 13.3)	0.06	10.6 (3.3, 17.9)	0.004
Time spent treating CTCL by respondent		<0.001		<0.001		<0.001		<0.001
<15 min per day	(ref)		(ref)		(ref)		(ref)	
16–60 min per day	23.3 (16.7, 29.8)		22.0 (14.6, 29.3)		24.9 (17.6, 32.2)		22.3 (14.4, 30.2)	
>60 min per day	25.9 (18.8, 33.1)		24.7 (16.6, 32.8)		23.5 (15.6, 31.5)		30.6 (21.9, 39.3)	
Burden of CTCL^b^								
Treated unfairly because of CTCL	26.7 (17.3, 36.2)	<0.001	15.2 (4.5, 25.9)	0.006	28.5 (18.2, 38.9)	<0.001	33.1 (22.0, 44.2)	<0.001
CTCL has been mistaken for a contagious condition	21.8 (15.3, 28.2)	<0.001	18.5 (11.2, 25.8)	<0.001	18.9 (11.7, 26.2)	<0.001	28.9 (21.4, 36.4)	<0.001
CTCL has to be kept private from others	15.4 (9.3, 21.5)	<0.001	5.9 (−1.0, 12.8)	0.091	17.6 (11.0, 24.3)	<0.001	20.2 (13.1, 27.4)	<0.001
Financially burdened by cost of managing CTCL	19.4 (13.5, 25.3)	<0.001	16.5 (9.8, 23.1)	<0.001	19.3 (12.8, 25.9)	<0.001	22.3 (15.4, 29.3)	<0.001
Any of the above	20.5 (13.1, 28.0)	<0.001	14.6 (6.2, 22.9)	0.001	20.9 (12.7, 29.2)	<0.001	25.4 (16.6, 34.1)	<0.001

Abbreviation: CTCL, cutaneous T‐cell lymphoma.

^a^ Regression coefficient, corresponding to the mean change in quality of life score per change in the associated variable.

^b^ Dichotomized as sometimes, often or very often versus rarely or never (reference); see Table 4, 5 for the text of the original question.

**TABLE 5:** Associations with Health‐Related Quality of Life as measured by the FACT‐G

	Total score		PWB subscale		SWB subscale		EWB subscale		FWB subscale	
Variable	β^a^ (95% CI)	*p*‐Value	β^a^ (95% CI)	*p*‐Value	β^a^ (95% CI)	*p*‐Value	β^a^ (95% CI)	*p*‐Value	β^a^ (95% CI)	*p*‐Value
Age, per 10‐year increase	1.9 (0.3, 3.6)	0.022	0.5 (−0.0, 1.1)	0.057	0.4 (−0.2, 1.0)	0.17	0.7 (0.3, 1.2)	0.002	0.2 (−0.3, 0.7)	0.41
Advanced stage (vs. early stage)	−11.1 (−17.6, −4.5)	0.001	−4.0 (−6.1, −1.9)	<0.001	−0.3 (−2.7, 2.2)	0.84	−1.6 (−3.5, 0.3)	0.11	−5.3 (−7.3, −3.3)	<0.001
Body area involved (vs. other area)										
Head and neck	−9.8 (−14.4, −5.2)	<0.001	−4.5 (−5.9, −3.0)	<0.001	−0.1 (−1.8, 1.5)	0.87	−2.3 (−3.6, −1.0)	0.001	−3.0 (−4.4, −1.5)	<0.001
Hands or feet	−9.2 (−13.7, −4.6)	<0.001	−3.9 (−5.3, −2.4)	<0.001	−0.6 (−2.3, 1.0)	0.46	−1.3 (−2.6, 0.0)	0.056	−3.7 (−5.1, −2.3)	<0.001
Groin	−9.6 (−14.9, −4.4)	<0.001	−3.6 (−5.3, −1.9)	<0.001	−1.0 (−2.9, 0.9)	0.3	−1.5 (−3.0, 0.0)	0.052	−3.1 (−4.8, −1.5)	<0.001
Itching severity, per 1‐point increase	−2.5 (−3.2, −1.7)	<0.001	−1.1 (−1.4, −0.9)	<0.001	−0.1 (−0.4, 0.2)	0.4	−0.4 (−0.7, −0.2)	<0.001	−0.8 (−1.0, −0.5)	<0.001
Time since diagnosis		0.54		0.12		0.18		0.3		0.25
<1 year	(ref)		(ref)		(ref)		(ref)		(ref)	
1–5 years	0.1 (−5.9, 6.1)		0.6 (−1.3, 2.6)		−0.8 (−2.9, 1.4)		1.1 (−0.6, 2.8)		−0.6 (−2.5, 1.3)	
>5 years	1.7 (−4.5, 7.9)		1.3 (−0.7, 3.4)		−1.4 (−3.6, 0.8)		1.3 (−0.4, 3.1)		0.7 (−1.2, 2.7)	
Help from caregiver (vs. no help)	−3.8 (−8.3, 0.7)	0.099	−2.3 (−3.8, −0.9)	0.002	3.0 (1.4, 4.5)	<0.001	−2.0 (−3.3, −0.8)	0.002	−2.4 (−3.8, −1.0)	0.001
Time spent treating CTCL by respondent		<0.001		<0.001		0.64		0.001		<0.001
<15 min per day	(ref)		(ref)		(ref)		(ref)		(ref)	
16–60 min per day	−10.5 (−15.5, −5.5)		−4.2 (−5.8, −2.7)		−0.6 (−2.5, 1.2)		−2.7 (−4.1, −1.2)		−3.2 (−4.8, −1.6)	
>60 min per day	−15.0 (−20.5, −9.4)		−6.5 (−8.2, −4.7)		−0.8 (−2.9, 1.2)		−2.8 (−4.4, −1.2)		−5.0 (−6.8, −3.3)	
Burden of CTCL^b^										
Treated unfairly because of CTCL	−16.0 (−22.8, −9.2)	<0.001	−5.1 (−7.4, −2.8)	<0.001	−3.0 (−5.5, −0.4)	0.022	−4.6 (−6.5, −2.6)	<0.001	−3.5 (−5.7, −1.2)	0.002
CTCL has been mistaken for a contagious condition	−12.5 (−17.2, −7.7)	<0.001	−4.8 (−6.4, −3.3)	<0.001	−2.4 (−4.2, −0.7)	0.007	−2.5 (−3.9, −1.1)	<0.001	−3.0 (−4.5, −1.5)	<0.001
CTCL has to be kept private from others	−11.5 (−15.9, −7.1)	<0.001	−2.0 (−3.4, −0.5)	0.01	−4.3 (−5.9, −2.7)	<0.001	−2.5 (−3.8, −1.3)	<0.001	−2.6 (−4.0, −1.2)	<0.001
Financially burdened by cost of managing CTCL	−13.7 (−18.0, −9.4)	<0.001	−4.5 (−5.9, −3.1)	<0.001	−2.5 (−4.1, −0.9)	0.002	−2.9 (−4.2, −1.7)	<0.001	−3.8 (−5.2, −2.4)	<0.001
Any of the above	−15.8 (−21.3, −10.4)	<0.001	−4.2 (−5.9, −2.4)	<0.001	−4.6 (−6.6, −2.7)	<0.001	−3.3 (−4.9, −1.7)	<0.001	−3.8 (−5.5, −2.0)	<0.001

Abbreviations: CTCL, cutaneous T‐cell lymphoma; EWB, emotional well‐being; FWB, functional well‐being; PWB, physical well‐being; SWB, social/family well‐being.

^a^ Regression coefficient, corresponding to the mean change in quality of life score per change in the associated variable.

^b^ Dichotomized as sometimes, often or very often versus rarely or never (reference); see Table 4, 5 for the text of the original question.

## 5 DISCUSSION

Overall HRQoL among our cohort of patients with CTCL as assessed by Skindex‐16 is similar compared to patients with other chronic dermatologic diseases, including eczematous dermatitis and psoriasis.^13^ Our cohort reported worse HRQoL as measured by FACT‐G compared to CTCL patients from previous studies by Demierre et al.^14^ The inclusion of the FACT‐G instrument in our survey also allows us to contrast our results with other cancer patients. Our cohort had worse HRQoL scores than long‐term survivors of indolent and aggressive non‐Hodgkin lymphoma.^15^ The mean total FACT‐G score in our patients was equivalent to cancer patients with an Eastern Cooperative Oncology Group Performance Status Rating (ECOG PSR) category of 2 (‘require bed rest for <50% of waking day’).^16^

As a disorder that spans chronic skin disease and cancer, the optimal instrument for assessing overall HRQoL for CTCL is not clear. There is variability in the existing CTCL HRQoL literature around instruments used, with most groups using the Skindex‐29,^1^, ^14^, ^17^, ^18^, ^19^ but FACT‐G^14^ and EORTC QLQ‐C30^18^ have also been used. We chose to use the shorter Skindex‐16 given the high validity in other skin diseases,^11^ which impacts the direct comparability of our numerical results but not trends or associations. We found similar associations and trends between the Skindex‐16 and FACT‐G. One important distinction is as a cancer‐specific instrument, the FACT‐G does not specifically assess skin symptoms such as itching. This was evident when comparing the overall HRQoL scores between patients with early and advanced disease across subscales, in particular, the symptoms subscale of the Skindex‐16, for which there was a nearly twofold difference in scores (29 and 50 for early and advanced disease, respectively) compared to a smaller difference in the physical well‐being subscale of the FACT‐G (23 and 20 for early and advanced disease, respectively). We recommend that when HRQoL of patients with CTCL is assessed using FACT‐G, additional questions around skin symptoms, particularly itch, are added. Alternatively, validation of a CTCL‐specific FACT instrument could address these concerns, while maintaining the benefits of comparability with the existing data.

Patients with more advanced CTCL had worse HRQoL compared to those with early disease in this study. The association with stage was significant for both the Skindex‐16 (*p* < 0.001) and FACT‐G (*p* = 0.001) instruments, even when adjusted for age, gender and number of medical comorbidities (data not shown). These results are consistent with clinical experience and prior studies, suggesting patients with later‐stage disease generally experience more symptoms and greater morbidity than those with early‐stage disease.^1^, ^7^, ^17^, ^19^ Demierre et al. reported that patients with more advanced CTCL reported more effects on general health, particularly in the physical, emotional and functional domains of the FACT‐G.^14^ In our cohort, patients with advanced disease had statistically significantly worse HRQoL on all of the Skindex‐16 subscales (symptoms, emotions and functioning), but only on two of the four FACT‐G subscales (physical and functional well‐being), suggesting that later disease stage most severely impairs patients' overall physical health and ability to perform daily tasks, while the emotional impact is high regardless of stage.

We hypothesized that patients who were newly diagnosed with their CTCL might experience worse HRQoL, positing that patients who had more time to acclimate to their diagnosis might fare better. This did not prove to be the case, with no significant difference in HRQoL between those diagnosed <1 year, 1–‐5 years or >5 years. We found that the time spent treating CTCL, in particular, spending >15 min daily, was associated with worse HRQoL. Spending >60 min daily treating CTCL had a large impact on HRQoL (Tables 4 and 5). Asking patients about the amount of time spent daily treating their CTCL may provide important insight into their disease‐related HRQoL.

The mean VAS itch score in our cohort was 3.2 on a 10‐point scale, representing moderate pruritus.^20^ Similar to others,^1^, ^17^ we found that higher itch scores were significantly associated with worse HRQoL on the Skindex‐16 (*r*_s_ = 0.56; *p* < 0.001) and FACT‐G (*r*_*s*_ = −0.36; *p* < 0.001) instruments. Existing literature supports that patients with chronic pruritus have lower HRQoL, and that pruritus may contribute to other symptoms (e.g., sleep disturbance, depression) which further compound the problem.^21^ It is important to note that our cohort had slightly lower VAS scores compared to a larger cohort of CTCL patients (mean of 4.2).^22^ This suggests our data might underestimate HRQoL in the general CTCL population.

Patients who reported involvement of their CTCL in the head/neck, hands/feet or groin/genitals had significantly worse HRQoL compared to those with other body parts affected (Tables 4 and 5). This association was seen globally and across all three Skindex‐16 subscales (*p* < 0.001 for each), as well as the global and physical and functional well‐being subscales on the FACT‐G (*p* < 0.001 for each) (Tables 4 and 5). Our findings add to those of the Prospective Cutaneous Lymphoma International Prognostic Index (PROCLIPI) study, which demonstrated a worse QoL in patients with alopecia.^19^

We found that nearly one‐quarter (24%) of patients had involvement of their CTCL in the groin/genitals. Previous studies have reported on the impairment on sexual life reported by patients with CTCL,^18^ and we confirm that worse HRQoL is reported by patients with groin/genital involvement by their CTCL. This finding of worse HRQoL with groin/genital involvement is not surprising when put in the context of the impact on HRQoL of other skin conditions that impact the groin/genitals. Groin/genital involvement in patients with hidradenitis suppurativa (HS) is associated with decreased HRQoL generally,^23^ and patients with HS report lower sexual health and sexual function.^24^ Our findings reinforce the importance of asking patients about and evaluating for the involvement of CTCL in the groin/genitals, as focusing on the treatment of these areas could significantly impact patient’s sexual health and overall HRQoL.

Based on the findings of Demierre's group,^7^ we were inspired to further evaluate the themes from the independent measures in that study along with one additional measure (Table 4, 5). The Burden Score questions address possible stigma or shame that patients with CTCL might experience (such as needing to keep the condition private and being treated unfairly because of CTCL). Collectively, the Burden Score was significantly associated with overall HRQoL by both instruments used, with an impact on par with the stage of disease (Tables 4 and 5). This suggests that patients with CTCL may experience significant emotional distress from shame and stigma associated with their disease which, when added to the impact of physical symptoms such as itching and poor sexual function, contribute to worse overall quality of life, and studies to further validate the Burden Score are warranted.

This study is one of the largest describing HRQoL in CTCL,^7^, ^17^, ^18^, ^19^ though there are factors which might limit the generalizability of our findings. Given the rarity of CTCL, this survey was distributed electronically via disease‐specific social media groups and email listservs with overlapping membership, making calculation of a response rate not feasible. Our sample was of a similar age (mean 57 years) to the average age of CTCL patients ^25^; however, our patient group had more who identified as white and female than the overall population of patients with CTCL^26^ (though in similar proportions to that of Demierre et al.^7^). There are known racial disparities in the age of presentation and outcomes of non‐white patients with CTCL^27^, ^28^, ^29^; given the low overall number of non‐white respondents in our group we could not assess racial disparities in HRQoL. Our sample also contains fewer patients with CD30 LPDs compared to the known frequency of these disorders,^25^ possibly because this group of patients may not be as active in support groups. Importantly, the data in this study are patient‐reported, and subject to bias both at the level of self‐selection and self‐reporting. Lastly, we did not ask about specific treatments for CTCL and cannot comment on the relationship of particular treatments to HRQoL.

## 6 CONCLUSIONS

Individuals with CTCL continue to be profoundly impacted by their disease. As a condition that has components of cancer and chronic skin disorder, patients with CTCL experience intense physical and psychosocial suffering across multiple domains which encompass negative social interactions, contributions by stigma and shame, and impacts on physical functioning including patients' sexual lives. Future research must focus on developing evidence‐based interventions that address these critical domains of everyday life to improve the quality of life for CTCL patients across their disease trajectory.

## CONFLICTS OF INTEREST

The authors have no conflicts of interest to disclose.

## Data Availability

The data that support the findings of this study are available from the corresponding author upon reasonable request.
